# Hummingbirds use distinct control strategies for forward and hovering flight

**DOI:** 10.1098/rspb.2023.2155

**Published:** 2024-01-10

**Authors:** Vikram B. Baliga, Roslyn Dakin, Douglas R. Wylie, Douglas L. Altshuler

**Affiliations:** ^1^ Department of Zoology, University of British Columbia, Vancouver, British Columbia, Canada V6T 1Z4; ^2^ Department of Biology, Carleton University, Ottawa, Ontario, Canada K1S 5B6; ^3^ Department of Biological Sciences, University of Alberta, Edmonton, Alberta, Canada T6G 2R3

**Keywords:** hummingbirds, flight control, manoeuvrability, stability, vision

## Abstract

The detection of optic flow is important for generating optomotor responses to mediate retinal image stabilization, and it can also be used during ongoing locomotion for centring and velocity control. Previous work in hummingbirds has separately examined the roles of optic flow during hovering and when centring through a narrow passage during forward flight. To develop a hypothesis for the visual control of forward flight velocity, we examined the behaviour of hummingbirds in a flight tunnel where optic flow could be systematically manipulated. In all treatments, the animals exhibited periods of forward flight interspersed with bouts of spontaneous hovering. Hummingbirds flew fastest when they had a reliable signal of optic flow. All optic flow manipulations caused slower flight, suggesting that hummingbirds had an expected optic flow magnitude that was disrupted. In addition, upward and downward optic flow drove optomotor responses for maintaining altitude during forward flight. When hummingbirds made voluntary transitions to hovering, optomotor responses were observed to all directions. Collectively, these results are consistent with hummingbirds controlling flight speed via mechanisms that use an internal forward model to predict expected optic flow whereas flight altitude and hovering position are controlled more directly by sensory feedback from the environment.

## 1. Introduction

Animals moving through their environment make use of multiple sensory cues to control their locomotion. A key visual signal is optic flow, defined as the movement of surfaces and edges from the environment across the retina due to self-motion. When optic flow signals are strong, these are used to drive optomotor responses and control ongoing locomotion [1].

The optomotor response is an example of a behaviour that is driven by an external stimulus, in which the behaviour cancels the stimulus that caused it. Hummingbirds have been shown to hold hovering position by using an optomotor response to counter experimentally induced optic flow [2]. When optic flow was displayed in front of a feeder, hummingbirds drifted in concert with each tested direction: upward, downward, left, right, looming and receding. The response persisted through time and was proportional to the magnitude of optic flow displayed in the visual field.

Forward flight is a behaviour that naturally induces optic flow owing to self-motion. Optic flow signals should not be cancelled as in an optomotor response because that would cause the bird to stop flying forward. Instead, forward flight requires that an internal model of the relationship between motor commands and their sensory consequences be used to predict the portion of any optic flow signal caused by the bird's self-generated flight commands. There are two known internal models for control of locomotion, termed ‘forward’ and ‘inverse’ [3]. A forward model uses the motor commands to predict the sensory signals, whereas an inverse model uses the sensory signals to control the motor commands. For a visual animal in forward flight, these two models can be explained using velocity of optic flow, which is sometimes referred to as ‘pattern velocity’. A forward model predicts an expected pattern velocity, whereas an inverse model sets a desired pattern velocity with adjustments to motor output to reach that desired sensory state.

There is abundant evidence that some insects and birds use pattern velocity to control forward flight, a result that is consistent with internal inverse models. One visual guidance challenge, termed ‘centring’, is when an animal navigates through a narrow passage to avoid collisions with the side walls. An inverse model based on pattern velocity predicts that an animal should choose a lateral position through the tunnel that balances pattern velocity cues on either side. The centring behaviour of honeybees [4,5] and budgerigars [6] has been shown to match this prediction. Control of forward flight velocity using an internal inverse model would predict that an animal would adjust its ground speed to reach a preferred optic flow velocity on the eyes. Forward flight control in honeybees [7], flies [8,9] and budgerigars [10] generally matches this prediction. Internal inverse models driven by pattern velocity have therefore been shown to guide both centring and forward velocity in both insects and birds.

A known exception to the use of pattern velocity in forward flight is the centring response of hummingbirds. When challenged to fly through a narrow flight tunnel, hummingbirds ignored pattern velocity cues and instead tended to fly away from larger features and towards smaller features [11]. It was hypothesized that the algorithm hummingbirds used was to balance the rate of expansion in the left and right visual fields.

The strategy hummingbirds use to control forward flight is currently unknown. In the hummingbird centring study [11], several pattern velocity treatments were tested, whereas dynamic manipulations of visual expansion would be required to determine if this centring strategy was based on an inverse or forward model, or on a combination of both. In addition, lateral expansion cues can be effective for avoiding collisions, but this signal lacks information about forward motion. Therefore, it is currently unknown which internal model(s) and which visual signals hummingbirds use to control forward flight velocity.

To gain insight into the hummingbird forward flight control strategy, we performed a large number of exploratory manipulations. We examined the flight trajectories of hummingbirds in a flight tunnel with projections on the frontal and lateral walls. Based on previous studies of forward flight in insects and birds, we hypothesized that forward flight velocity in hummingbirds would be associated with either pattern velocity or rate of expansion cues. Pattern velocity cues included static and moving gratings. Expansion cues were tested through static horizontal gratings of different spatial frequencies. We further explored the effects of frontal visual cues using spirals that either loomed or receded. We were able to compare the effects of the same set of cues on hovering flight because hummingbirds frequently made voluntary pauses. The goal of this suite of manipulations was to derive a new hypothesis for flight control in hummingbirds.

## 2. Methods

Experiments were performed with eleven adult male Anna's hummingbirds (*Calypte anna*) that were captured from the wild using box traps. Birds were housed in 0.6 × 0.6 × 0.9 m chambers and provided with ad libitum access to artificial nectar (Nektar-Plus, Nekton, Pforzheim, Germany). All work was performed at the University of British Columbia and the procedures were approved by the institutional Animal Care Committee.

**Figure 1.**
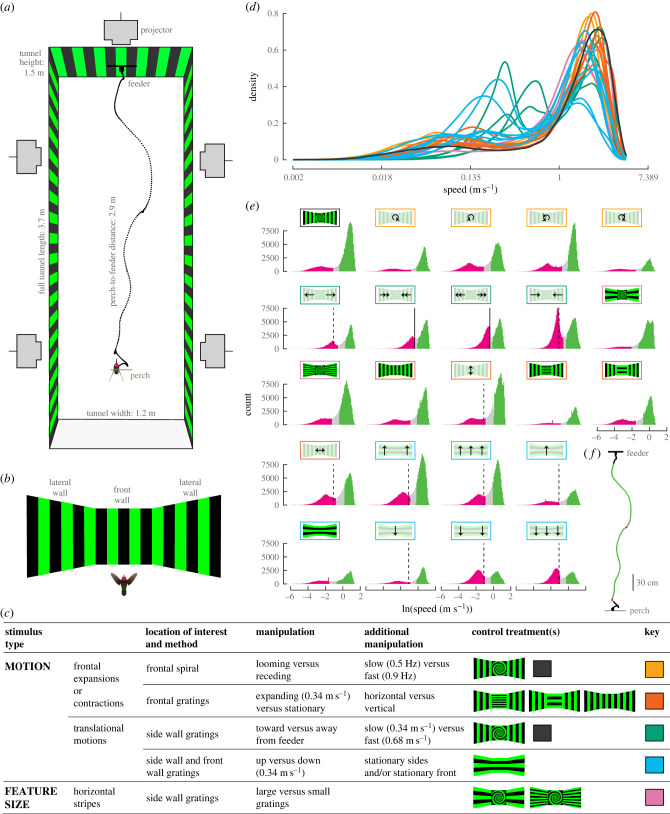
A flight chamber with multiple visual displays allowed testing the effects of flight mode (hovering versus forwards) and visual field (frontal versus lateral) on hummingbird position and velocity. (*a*) The chamber was 3.7 m long by 1.2 m wide by 1.5 m high. Five digital light processing (DLP) projectors displayed stimulus patterns on the walls. A representative trajectory is shown in black. (*b*) Each treatment is represented by a bird's perspective from the perch looking at the front and side walls. The patterns indicate arrangement only and are not to scale. (*c*) Flights were measured for 23 distinct stimulus treatments that varied in features and motion on the frontal and lateral walls. The control treatment for a static spiral on the front wall and static horizontal gratings on the side walls is indicated by a black square. Other control treatments with no motion are also indicated by schematics and the motion treatments associated with each control are indicated by colour key. (*d*) Flight speed was bimodally distributed across all treatments, but some treatments differed in the location of each peak. (*e*) The speed distributions for each treatment are shown, colour-coded by hovering (magenta), forward (green) or undefined (grey) frame sequences. For treatments with grating motion, slow (0.34 m s^−1^) and fast (0.68 m s^−1^) stimulus speeds are indicated by dashed and solid vertical lines, respectively. The schematics of control treatments with static stimuli are shown with full opacity and motion treatments are shown with lower opacity. (*f*) The trajectory in (*a*) is replotted with frame sequences coloured as either hovering (magenta), forward (green), or undefined flight (grey).

The experimental chamber was 3.7 m long by 1.2 m wide by 1.5 m high and constructed with an 80/20 aluminium frame and acrylic walls (figure 1*a*). The walls were covered with a white film (Wallpaper for Windows), which served as the surface for five ultrashort-throw, rear-mounted digital light processing (DLP) projectors (NEC U310W; 120 Hz refresh rate). A strip of near-infrared LED lights lined the top of the walls, and the top of the chamber was covered in opaque acrylic that passed infrared wavelengths.

Six charge-coupled device (CCD) cameras (Prosilica GE680, Allied Vision Technologies; Computar H2Z0414C-MP lenses) were positioned above the chamber. The cameras recorded at 100 frames per second and Flydra image-based tracking software [12] automatically tracked flight trajectories and computed the birds' three-dimensional position (*x*, *y* and *z* coordinates) in each frame. An example trajectory is shown in figure 1*a*.

To determine if forward flight speed is influenced by lateral and frontal visual stimuli (figure 1*b*), we performed five experiments (figure 1*c*). The goal of these experiments was to determine how different visual stimuli influence the control of forward flight velocity. We did not attempt to study learning across experiments so there was no (experimental) control over an individual's experience across the different experiments. Instead, we randomly ordered treatments within an experiment to ensure that treatment effects and order effects were not confounded. Prior to starting an experiment, we first verified that a bird could perform regular perching and dock at the feeder within the chamber under the control treatment for that experiment. Verification for a bird's very first experiment generally occurred within an hour. Stimulus treatments for an experiment were displayed for 20–30 min in a randomized order within each recording session. Each experiment included at least seven hummingbird subjects, with recording sessions for one to three experiments occurring for a given subject on a given day.

All stimuli were created using Psychtoolbox-3 for MATLAB (Mathworks), which also served to synchronize the displays. Side-wall patterns included green and black square-wave gratings that were oriented either vertically or horizontally and either moving or stationary. The grating stimulus period was either 9.2 or 1.15 cm, and the pattern velocity was either stationary (0 m s^–1^), ‘slow’ (0.34 m s^–1^) or ‘fast’ (0.68 m s^–1^). Forward motion was defined as toward the feeder whereas backward motion was toward the perch. The frontal patterns were either vertical or horizontal square-wave gratings, or spirals. The spiral was a four-armed 10° logarithmic spiral, constructed to match the spiral used in Goller & Altshuler [2]. The spiral pattern was either held stationary (0 Hz), ‘slow’ (0.5 Hz) or ‘fast’ (0.9 Hz). Looming motion was counterclockwise and receding motion was clockwise. We did not use white stimuli because the DLP projectors cycle through red, green and blue at 120 Hz using variable colour sequences to generate different colours. White is generated by cycling through red, green and blue equally, which creates a colour flicker at high speed. In our previous forward flight study, we used red and black patterns. In the current study, we used black and green, because green had higher luminance.

From the *xyz* tracking data, we measured the position, overall speed, and per-axis velocity through time [13]. An initial analysis revealed that speed distributions varied by treatment (figure 1*d*). Speed was bimodally distributed in every treatment: the slower peak values ranged from 0.10 to 0.62 m s^−1^, whereas the faster peak values ranged from 1.45 to 2.31 m s^−1^. These bimodal distributions indicated that hummingbirds were using both forward and hovering flight during experiments, and that the ranges of values within each of these two categories were affected by the treatment. We determined appropriate speed bounds for each flight mode within each treatment using finite Gaussian mixture models [14,15] (figure 1*e*). We omitted data from within 17 cm of the perch or feeder for two reasons. First, accelerations and/or tracking errors were higher in these regions. Second, our goal was to focus on forward and hovering flight rather than on take-offs and decelerations for docking at the feeder. The analysis revealed that forward flight could reliably be distinguished (posterior probability >95%) at speeds above a threshold of 0.5 to 1.33 m s^–1^, depending on treatment, whereas hovering could be distinguished (posterior probability >95%) by instances where the speed threshold was below 0.22 to 0.7 m s^–1^, again depending on treatment. Tracking sequences that could not be identified as either hovering or forward flight are indicated in grey in figure 1*e*. These data were omitted from all analyses to avoid conflating hovering with possible forward flight. The example trajectory from figure 1*a* is replotted with sequences of forward flight (green), hovering (magenta), or in-between (grey) in figure 1*f*.

**Figure 2.**
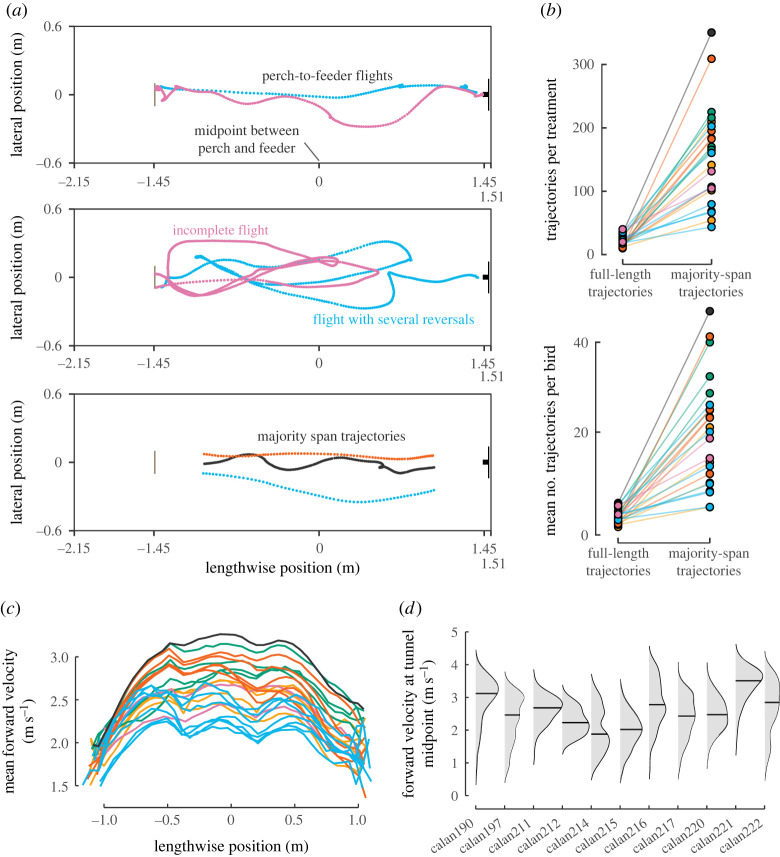
Variation in flight trajectories within the tunnel. (*a*) Example trajectories of perch-to-feeder flight (top) and of an incomplete flight and a flight with several reversals (middle) are shown. (*b*) Perch-to-feeder flights were rare, so we focused on flights that spanned most of the tunnel length (*a* bottom), which are much more numerous. (*c*) The average of the instantaneous forward velocities is shown colour-coded by treatment (as in figure 1*c*) for each lengthwise position of the majority-span flights. (*d*) The distribution of velocities at the mid-tunnel position (0 m in *c*) is shown for each of the 11 hummingbirds in the study. Horizontal bars indicate the median velocity.

We initially defined ‘full-length trajectories' as flights that started from 17 cm past the perch and spanned most of the length of the tunnel, up to 17 cm in front of the feeder. However, we found that this definition of full-length trajectories led to low and uneven sample sizes across stimulus treatment sets (figure 2*a*,*b*). Many forward flights did not span the full region of interest because hummingbirds exhibited changes in velocity or direction, i.e. manoeuvres. They sometimes reversed course and returned to the perch or paused their trajectory, transitioning from forward flight to hovering. Full-length trajectories represented less than 7.4% of all tracked data from flight sequences. We therefore elected to focus on the centre section of the tunnel (middle two-thirds = 2.4 m), which we term ‘majority-span trajectories’. This definition results in a much larger sample size of trajectories, especially across treatments.

Forward flight velocity (i.e. the component of speed along the lengthwise axis) varied from 0.3 to 4.5 m s^–1^ within the centre section. This variable flight velocity led to uneven sampling of frames within and between flight trajectories. To ensure evenness of sampling for statistical analysis, the centre section of the tunnel was divided into 40 bins along the length axis, and the position and velocity data within each bin were averaged for each flight trajectory. We tested hypotheses regarding the effects of visual stimuli on forward flight velocity using generalized additive mixed models (GAMMs) [16,17]. Because position in the tunnel could affect perception of visual stimuli, our candidate models included various combinations of positional data. Our choice to use GAMMs was motivated by three factors: (1) lengthwise position was observed to have a strongly nonlinear relationship with forward flight velocity (figure 2*c*), which would violate assumptions made by linear models, (2) individual birds varied in flight velocity (figure 2*d*), and (3) trajectory data involves the use of repeated measures. Data from all stimulus treatments were used in each model, and forward flight velocity (*V_x_*) was the dependent variable in all cases. Independent variables included combinations of stimulus treatment (*S*), identity of each bird (*B*), mean lengthwise position (*X*), mean lateral position (*Y*) and mean altitudinal position (*Z*):

2.1 $$\begin{array}{c} V_{x}=B+(1|T) \end{array},$$

2.2 $$\begin{array}{c} V_{x}=S+B+(1|T) \end{array},$$

2.3 $$\begin{array}{c} V_{x}=S+Y+Z+s(X)+B+(1|T) \end{array},$$

2.4 $$\begin{array}{c} V_{x}=S+Y+s(X*S)+B+(1|T) \end{array}$$

2.5 $$\begin{array}{c} \mathrm{and}\;V_{x}=S+Y+Z+s(X*S)+B+(1|T) \end{array}.$$

A smoothing term (*s*) was applied to *X* to accommodate nonlinearity in its relationship with *V_x_*. In addition, the identity of each trajectory (*T*) was used to inform random effects (i.e. repeated measures). To determine the best-fitting model, corrected Akaike information criterion (AICc) and Bayesian information criterion (BIC) were used.

We also asked how visual stimuli affected altitudinal position. The statistical approach for this question was like the approach for forward flight, but with altitudinal position (*Z*) as the dependent variable:

2.6 $$\begin{array}{c} Z=B+(1|T) \end{array},$$

2.7 $$\begin{array}{c} Z=S+B+(1|T) \end{array},$$

2.8 $$\begin{array}{c} Z=S+s(X)+B+(1|T) \end{array},$$

2.9 $$\begin{array}{c} Z=S+Y+s(X)+B+(1|T) \end{array}$$

2.10 $$\mathrm{and}\;\begin{array}{c} Z=S+Y*S+s(X)+B+(1|T) \end{array}.$$

We next focused on hovering flights following the pauses in forward flight and asked how visual stimuli affected hovering behaviour. Because pauses were frequent, we relaxed the requirement of position needing to be in the centre segment of the tunnel, and instead included all data that were at least 17 cm away from a perch or feeder. We then isolated segments with flight velocities identified as hovering (figure 1*e*) that lasted longer than 0.35 s. To determine if different visual stimuli influenced the onset location (*X*_0_, *Y*_0_ and *Z*_0_, for lengthwise, lateral and altitudinal, respectively) and duration (*D*) of hovering flight pauses, we analysed these data jointly by treatment and/or bird identity using linear mixed models [18]:

2.11 $$\begin{array}{c} \left(X_{0},Y_{0},Z_{0},D)=(1|B\right) \end{array}$$

and

2.12 $$\begin{array}{c} \left(X_{0},Y_{0},Z_{0},D)=S+(1|B\right) \end{array}.$$

**Figure 5.**
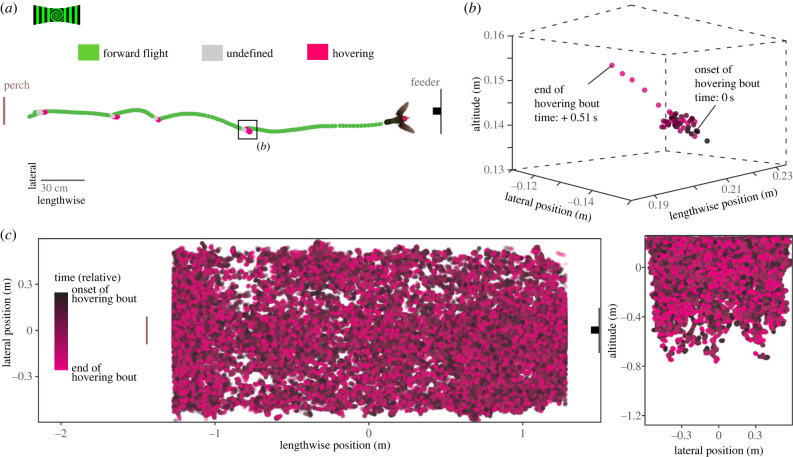
Hovering pauses in forward flight occurred frequently throughout the upper half of the chamber. (*a*) A representative trajectory from the perch to the feeder in which the animal voluntarily paused four times to hover. This trajectory is from a control treatment. The region within 17 cm of the perch and feeder is excluded. (*b*) A zoomed-in view of the pause indicated by the black box in (*a*). Colour indicates time, starting with the onset (dark) and progressing to the end (magenta, normalized) of the hover bout. (*c*) All hovering bouts in the study are shown in both a top view (lateral versus lengthwise position) and cross-section view (altitude versus lateral position) of the chamber.

To next determine if different visual stimuli influenced the direction of drift during hovering, we first standardized each hovering segment to begin at a position of (0, 0, 0) by subtracting the coordinates of the first frame of data (i.e. (*X*_0_, *Y*_0_, *Z*_0_)) from that of each subsequent frame within that segment. This allowed us to determine how hummingbirds drifted after the onset of hovering behaviour. We then tested hypotheses of the effects of visual stimuli on drift velocity in all three axes jointly ({*V_X_*, *V_Y_*, *V_Z_*}) using linear mixed models. Independent variables included combinations of stimulus treatment and onset position. Random effects included bird identity and trajectory identity:

2.13 $$\begin{array}{c} \left(V_{X},\;V_{Y},\;V_{Z})=(1|B)+(1|T\right) \end{array},$$

2.14 $$\begin{array}{c} \left(V_{X},\;V_{Y},\;V_{Z})=S+(1|B)+(1|T\right) \end{array}$$

2.15 $$\mathrm{and}\;\begin{array}{c} \left(V_{X},\;V_{Y},\;V_{Z})=S+X_{0}+Y_{0}+Z_{0}+(1|B)+(1|T\right) \end{array}.$$

## 3. Results

We recorded 7150 flight trajectories within the centre section of the tunnel (majority-span flights), including both perch-to-feeder and feeder-to-perch flights. The feeder side of the flight chamber included a stimulus screen, but the perch side did not (figure 1*a*). Because our aim was to test the hypothesis that frontal and lateral visual stimuli influence forward flight velocity, we only analysed the 3575 perch-to-feeder trajectories.

The best-supported model of forward flight velocity was equation (2.5), which included all these effects (electronic supplementary material, table S1). In addition to the stimulus treatments, the position of the hummingbird along each of the three axes of the chamber was important to explain variation in forward flight velocity. Position along the lengthwise axis had an interactive effect with treatment, whereas the effects of lateral distance and altitudinal position were more uniformly negative, regardless of treatment (electronic supplementary material, figure S1). Variation in forward flight velocity was also explained by individual bird identity, with mean effect ranging from −0.57 to 0.58 m s^–1^, indicating that individual birds could vary in average flight velocity by upwards of approximately 1 m s^–1^.

**Figure 3.**
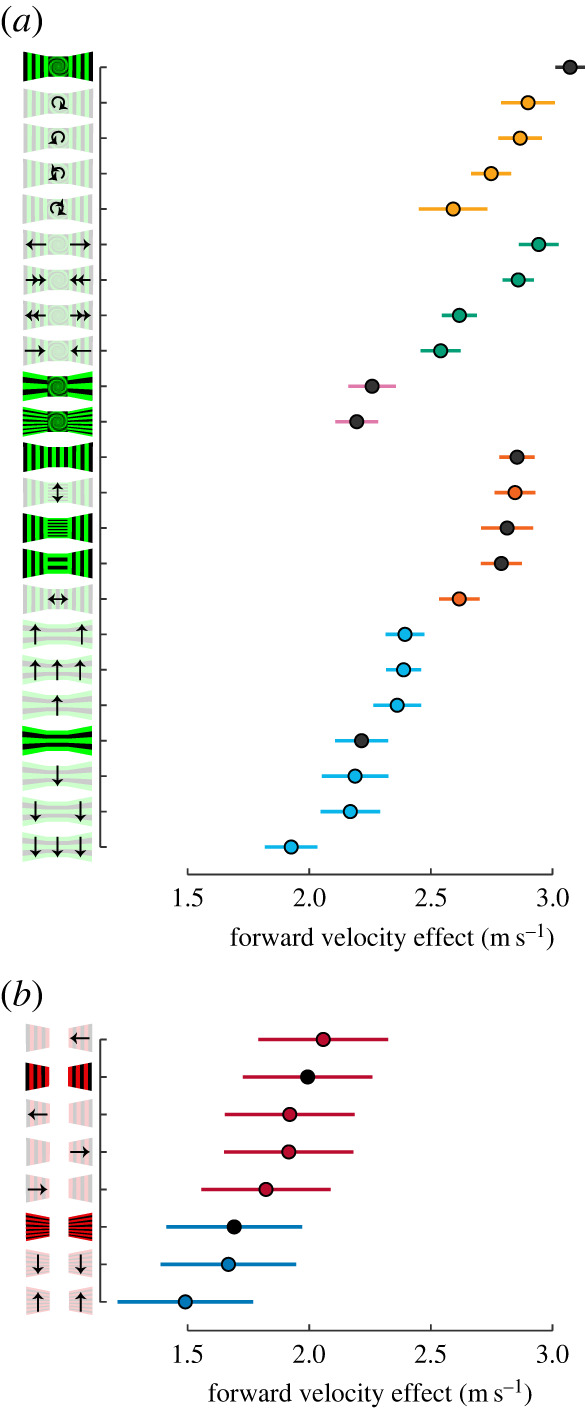
Forward flight velocity declined for visual stimuli that disrupted pattern velocity cues. (*a*) The forward velocity effects, after accounting for position in the tunnel and individual bird trajectory identities, are shown for all 23 treatments. Control treatments with static stimuli are shown with full opacity, and motion treatments are shown with lower opacity. Treatments with either stimulus motion or static horizontal gratings (i.e. that convey no forward pattern velocity) on the side walls resulted in reduced forward velocity. Circles indicate means and lines indicate 95% confidence intervals. (*b*) This result led us to analyse the velocity data from a previous study with a narrower chamber, which used asymmetrical side-wall patterns to test for stimulus effects on centring [11]. The narrower chamber led to slower velocities overall, but this analysis also supports the observation that reliable pattern velocity cues, specifically a static vertical grating on at least one side wall, generally led to faster forward velocity.

The forward velocity effect from the best-supported model is shown for all 23 treatments in figure 3*a*. We present the ‘forward flight velocity effect’ rather than the forward velocity because the former value is what remains after accounting for the effects of three-dimensional position within the chamber, individual trajectory, and individual bird. The control treatments are indicated by black circles and defined as having all stimuli held statically on the front wall and both side walls. The schematics for these treatments are shown with full opacity for emphasis. For experimental treatments, arrows on the lower-opacity schematics indicate the direction and strength of motion for gratings and spirals.

The most distinct effect of stimulus on forward velocity was the orientation of gratings on the side walls. Hummingbird flew faster in all treatments with vertical gratings than with horizontal gratings (figure 3*a*). The division seems to fall around a velocity of 2.4 m s^–1^ for this tunnel configuration. This unexpected result led us to re-examine the data collected from our earlier study, Dakin *et al*. [11], which was conducted in a narrower chamber and had the goal of determining the visual guidance for centring in hummingbirds. Dakin *et al*. did not use stimuli on the front wall, and the two side walls often contained different stimuli. However, our reanalysis also suggests that hummingbirds flew faster when side walls had vertical gratings (figure 3*b*). We also note that the velocities are slower, which we believe derived from the much narrower chamber (0.6 m versus 1.2 m in the current study). Moreover, that study had lower sample size (specifically number of trajectories) and wider 95% confidence intervals on mean velocity estimates.

A second important effect was that stimulus motion generally caused the birds to fly more slowly. This can be seen most clearly for the treatments with vertical gratings on the side walls and a spiral on the front wall. For these cases, any combination of motion caused the birds to fly more slowly than in the control (static) treatment. For the treatments in which the side walls had stationary vertical gratings and the front wall had either horizontal or vertical gratings (static or expanding), the birds flew at a similar speed. A key difference in front-wall stimuli is that approaching a spiral will produce information about expansion in all directions, whereas approaching a grating will produce information in only one direction, either vertical or horizontal. This apparent ambiguity from the gratings seems to cause hummingbirds to fly more slowly.

**Figure 4.**
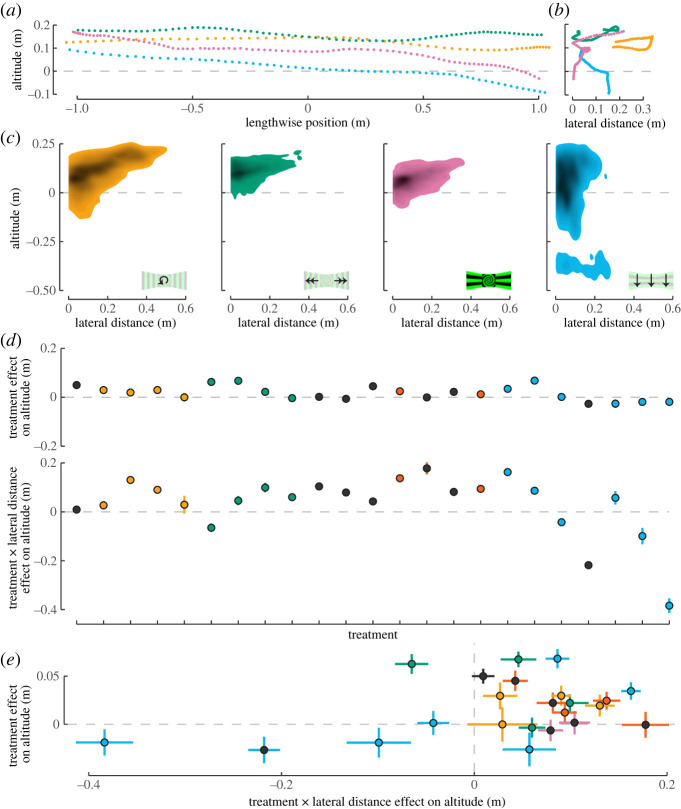
Vertical optic flow stimuli induced corresponding changes in altitude during forward flight that were amplified by proximity to the stimulus. Four example trajectories from different treatments are shown here, plotting altitude versus (*a*) length position and (*b*) lateral distance. (*c*) Two-dimensional kernel densities for whole populations depict altitude versus lateral distance for the same four treatments. Darker colours indicate higher densities of trajectories. (*d*) All 23 treatments analysed in a single mixed model. The effects of stimulus treatment on altitude are shown in the upper panel. Bars indicate 95% confidence intervals. The order of treatments (left to right) matches the order displayed in figure 3*a*, top to bottom. Effects on altitude due to the interaction between stimulus treatment and lateral distance are shown below. (*e*) The two effects from (*d*) are plotted against each other, illustrating the gain in response due to wall proximity.

We next examined the effects of optic flow on forward flight altitude. Figure 4*a* depicts example raw trajectories from the tunnel height versus tunnel length perspective from four different treatments. Birds maintained altitude in some cases and changed altitude in others. The change in altitude with lateral distance for the same example trajectories is shown in figure 4*b*. Examining the two-dimensional kernel density plots for the full population data from three of the four trajectories illustrates a general trend: trajectories that deviated farther from the tunnel midline (increased lateral distance) tended to be associated with higher altitude (figure 4*c*). A notable exception is the treatment with horizontal gratings moving downward (blue). In this case, deviation from the midline led to a decrease in altitude.

The best-supported model to explain variance in altitude included individual and trajectory identification, lengthwise position in the tunnel, treatment and the interaction between treatment and lateral distance (equation (2.10); electronic supplementary material, table S2). Figure 4*d* shows both the treatment effect (above) and the treatment by lateral distance effect (below) on altitude for all 23 treatments. These same effects are plotted against each other (figure 4*e*), which shows that the effects of stimulus on altitude became stronger as a bird deviated from the midline and towards the stimulus.

Birds tended to fly above perch height (dashed horizontal lines in figure 4). The convention for these plots is that 0 altitude represents perch height. The tunnel height was 0.25 m above the perch, which physically limited the upper altitude. In all the treatments with horizontal bars that were either stationary or moving down, the birds descended below the perch altitude. In the other treatments with horizontal bars (blue), birds ascended to a higher altitude when the side walls were moving upward but flew generally close to perch altitude when the pattern on the frontal wall was moving up or down.

Pauses in forward flight that led to hovering were common and could occur in any part of the upper half of the chamber (figure 5). The average number of pauses per forward flight trajectory ranged from 2.0 to 6.3, depending on treatment (range with 95% CI: 1.0 to 7.4 pauses; electronic supplementary material, figure S2). Some treatments tended to cause more frequent pauses, but the variation was large overall. On average, hovering bouts lasted between 0.83 and 1.03 s (range with 95% CI: 0.48 to 1.76; electronic supplementary material, figure S3). In some treatments, hovering bouts occurred more frequently near the feeder and/or perch or more laterally. However, there was no strong association between type of stimulus and average hovering bout location (electronic supplementary material, table S3).

After birds had transitioned from forward flight to hovering, the diverse stimuli could in principle cause drift in all three major axes. However, because the specific stimuli were designed to examine the influence of optic flow on forward flight velocity (figure 3) and altitude (figure 4), the stimuli differed primarily in the length and height components. All hovering sequences, relative to their starting position, are displayed for six representative treatments (figure 5*a*). During the control treatment, hovering hummingbirds held their lengthwise position, lateral position, and altitude. During the treatments with stationary spirals in front and moving vertical gratings on the sides (green traces in all figures), hovering hummingbirds tended to drift more in all directions, relative to control treatments. However, they drifted strongly in the same direction as the side-wall stimulus. For a treatment with stationary vertical gratings on the side walls and front-wall motion (orange traces), frontal expansion also caused hovering hummingbirds to drift backward in lengthwise position, away from the front wall. For treatments with horizontal gratings on all walls (blue traces), hovering birds drifted upward when the stimuli moved upward but they reached a ceiling both figuratively and, in many cases, literally. By contrast, downward stimulus motion caused greater magnitude of downward drift.

**Figure 6.**
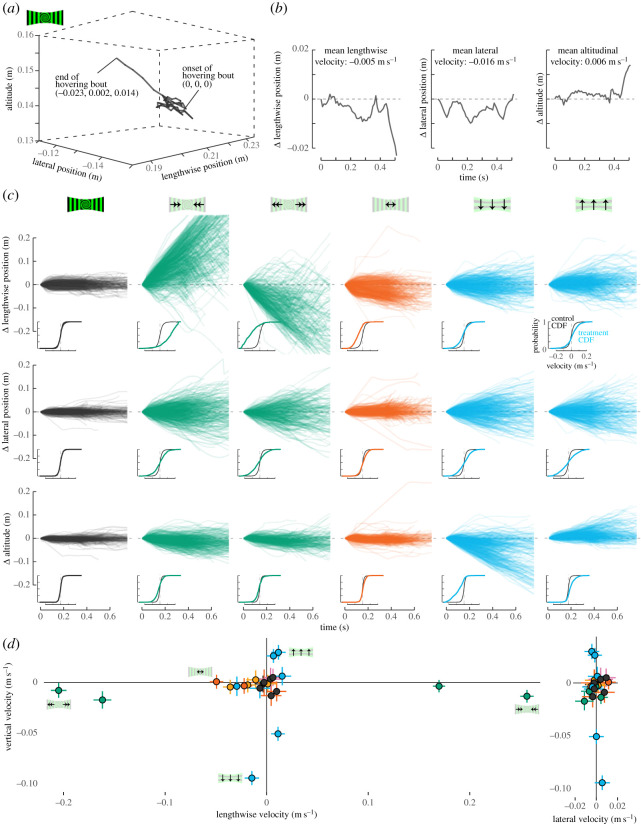
During hovering bouts, hummingbirds drifted most strongly in the direction of the stimulus motion that was displayed. (*a*) The same hovering bout from figure 5*b* is shown with onset-subtracted coordinates. (*b*) Relative change in position along each of the three axis is shown along with the mean velocity in each dimension. (*c*) All drifts through time are shown for each of six treatments (icons above each column) for all three axes. All bouts are aligned such that 0 time was the onset of hovering and are limited to display only the first 0.75 s. Note that the ranges of the *y*-axes in (*c*) are much larger than in (*b*) to account for larger drifts in treatments with moving patterns. Insets show the cumulative distributions of velocity through time for each axis. Black traces show the distribution pooled for all seven control treatments. Coloured traces indicate the distribution for each experimental treatment. (*d*) The mean effects (circles) and 95% confidence intervals (lines) for vertical velocity versus lengthwise velocity (left) and lateral velocity (right) are shown for all 23 treatments. Icons are included in the left panel for the non-control treatments in (*c*). CDF: cumulative distribution function.

During drifts, the change in position through time along each axis provides the axis-specific velocity. Insets underneath the traces in figure 6*c* show the cumulative probability distribution of each velocity component. The black curves represent the average for all static controls whereas the coloured curves indicate the values for each treatment and axis. A left or right shift in the distribution relative to the control indicates more negative or more positive velocities, respectively, for a given axis. The largest left–right shifts in figure 6*c* are observed in the green and blue traces for the dimension of stimulus motion. A shift to a shallower slope, compared with the control, indicates greater spread in velocity values. Shallower slopes are a more general feature in most axes for experiments with side-wall motion.

The effect of stimulus on vertical, lengthwise, and lateral velocities for all 23 treatments shows the strong effects of side-wall stimulus in either the vertical or lengthwise axis (figure 6*b*). The best-supported model to explain variation in drift velocity was equation (2.15) (electronic supplementary material, table S4). This model included the effects of stimulus treatment, onset position, bird identity, and trajectory identity. All the control treatments (black filled circles) resulted in close to 0 velocity in all axes (figure 6*d*). Nearly all the treatments with front-wall motion (yellow–orange and red–orange filled circles) caused the birds to drift away from the front wall (negative in the lengthwise velocity axis). None of the treatments caused consistent changes in lateral velocity. In the treatments with vertical gratings in motion on the side walls (green circles), two velocities were tested, and the faster velocity stimulus tended to cause faster velocity drifts. The most distal points are the faster velocity treatments. A related effect of stimulus strength can be seen for the treatments with horizontal gratings moving downward. The treatment with downward motion on the front wall and side walls caused higher downward velocity than the treatment with only side-wall motion. Because hummingbirds already flew near the top of the chamber, the treatments with upwards stimuli did not cause as strong an effect.

## 4. Discussion

We designed 23 treatments to determine how lateral and frontal optic flow signals influenced hummingbird forward flight velocity and altitude (figure 1*a–c*). Within each treatment, the left and right side walls had identical displays. Hummingbirds exhibited a diversity of flight behaviours in the tunnel, including hovering, manoeuvring, and forward flight (figures 1*d–f* and 2). Forward flight velocity was fastest when hummingbirds were presented with a control (static) treatment with vertical bars on the side walls and a spiral on the front wall (figure 3). All other treatments disrupted the hummingbirds' ability to estimate forward flight velocity from optic flow cues and caused them to fly slower. There were no coherent effects of either the direction or velocity of stimulus motion on forward flight velocity. However, hummingbirds flew slowest when the side walls had horizontal gratings, which have zero pattern (optic flow) velocity along the forward–backward axis. We also examined altitude control during forward flight and found that ascending horizontal gratings caused hummingbirds to fly near the top of the chamber, whereas descending or static horizontal gratings caused them to fly lower relative to other treatments and generally below perch level (figure 4). Pauses in forward flight that led to hovering could occur anywhere in the chamber regardless of treatment (figure 5; electronic supplementary material, figure S3). Unlike in forward flight, the direction and speed that hummingbirds drifted during hovering were strongly related to the direction and speed of side-wall stimulus motion (figure 6). These results suggest that during forward flight, hummingbirds look to the external environment for verification of an internal prediction of their forward velocity, whereas during hovering, they use sensory feedback from the external environment to guide their holding position. Altitude control during forward flight resembles hovering, insofar as it is like an optomotor response.

Visual guidance of hovering and forward flight in hummingbirds has been investigated separately in three previous studies. Two of these studies focused on hovering and found that hummingbirds tracked direction [2] as well as speed [19] of optic flow stimuli. The current study extends this result by examining hovering following transitions from forward flight. The third study focused on centring during forward flight, i.e. when an individual adjusts its lateral position through a narrow passage in response to asymmetrical visual stimuli on the left and right walls [11]. Hummingbirds did not centre in response to moving vertical gratings, but instead avoided large horizontal gratings (low spatial frequency), which caused high rates of vertical expansion. Our reanalysis of data from Dakin *et al*. [11] to examine forward flight velocity also suggests that hummingbirds flew slower with horizontal as opposed to vertical gratings on the side walls. Thus, both the previous and the current study demonstrate that horizontal bars, which eliminate forward-oriented optic flow velocity cues, are associated with a reduction in hummingbird forward flight velocity.

The responses we observed in hummingbirds during forward flight are inconsistent with direct reliance on sensory feedback from the environment. Evidence consistent with internal inverse models to control forward flight speed has been observed in honeybees [5], flies [8,9] and budgerigars [20]. Instead, hummingbirds flew fastest when the tunnel stimuli provided a reliable signal of optic flow that was consistent with the sensory feedback predicted by their self-generated action of forward flight. All manipulations of optic flow, either through changing lateral velocity, through eliminating lateral velocity, or through frontal expansion or contraction, created a mismatch between what a bird sensed and the optic flow signal predicted from self-motion. These results are consistent with hummingbirds using an internal forward model to guide forward flight speed.

Birds should require an internal model to control flight speed because a simpler controller based on only external signals, such as a proportional-integral-derivative (PID) controller, will have low gain and slow feedback [3]. Internal models are able to capture complex dynamics, anticipate the effects of muscle activity, and generate corresponding control signals. Of the two known internal models, the forward model has lower feedback delay. This is because the internal feedback signal is rapidly available, and even faster than the actual feedback signals resulting from movement. In principle, a forward model is a near-optimal feedforward controller because, without feedback delay to the motor system, the control function can have high gain [21].

There is accumulating evidence that the vertebrate cerebellum is a brain region that contains internal models, with some experiments providing support for inverse models and others for forward models [3]. There is also evidence that both models can be combined into a single tandem model for some types of human motor learning [22].

There is a rapid pathway for optic flow signals to reach the cerebellum. Optic flow is first computed in two retinal recipient areas in the midbrain of tetrapods: the pretectum and the accessory optic system [23–25]. In birds, these pretectal and accessory optic nuclei are known, respectively, as the lentiformis mesencephali (LM) and the nucleus of the basal optic root (nBOR) [26]. As in other tetrapods, in most birds the direction of optic flow is parsed between these two sites. The majority of LM neurons respond to temporal-to-nasal stimulus direction, whereas the other three cardinal directions are encoded by separate populations of nBOR neurons [27–29]. For altitude control, the populations of neurons in the nBOR that prefer upward and downward visual motion will influence these pre-motor neurons to generate movement to minimize optic flow. These same nBOR neurons as well as neurons preferring backward (nBOR) and forward (LM) visual motion would together provide the visual signals to control hovering along any axis in three-dimensional space [2].

In hummingbirds, the LM shows a massive hypertrophy compared with that of other birds [30]. Moreover, hummingbirds are unusual in that their LM neurons prefer faster velocities and have a broader distribution of direction preferences, although still with many forward-preferring neurons [27,28]. Furthermore, in both the LM and nBOR of hummingbirds, the neurons are more narrowly tuned in the spatio-temporal domain [28,29], indicating an enhanced capacity to encode optic flow speed at high resolution.

LM and nBOR project to two regions of the cerebellum, the vestibulocerebellum and the oculomotor cerebellum [31–37]. Both regions are expected to have a role in flight control, but the projections to the oculomotor cerebellum suggest it may be especially well suited as the site of an internal forward model that requires a copy of the motor signal [38]. Specifically, optic flow signals from LM and nBOR in the oculomotor cerebellum can be integrated with descending projections related to motor planning and execution, originating in the nidopallium [39,40] and the anterior wulst [41] of the telencephalon. Thus, a copy of a motor command (efference copy) can be processed at this site via a forward model to predict the expected sensory consequences of that command, which is then compared with actual sensory feedback.

Efference copies are challenging to demonstrate experimentally and there are no direct measurements of them in animal flight. However, experiments with tethered fruit flies (*Drosophila melanogaster*) demonstrate motor-related suppression of optic flow signals that would otherwise cause deleterious (destabilizing) optomotor responses, and lack of suppression for signals that cause useful (stabilizing) optomotor responses [42]. The behavioural results of the current study suggest that hummingbirds lack motor-related inputs to optic flow neurons during hovering and slow flight. By contrast, during forward flight they will have some comparison between motor-predicted optic flow and visually measured optic flow. Specifically, hummingbirds are predicted to suppress optic flow velocity that matches their intended velocity. In this neural model, any other flow velocity would be registered by the midbrain optic flow pathway [43] and lead to interruption of behaviour.

An internal forward model can generate a predicted sensory signal for control of fast movement, and can also be used to compute prediction error for motor learning [44]. This feature leads to a testable behavioural prediction of our hypothesis that hummingbirds are using an internal forward model for forward velocity control. If presented persistently with manipulated optic flow that is inconsistent with the optic flow predicted based on the bird's flight speed, over time the bird should compensate for this inconsistency by learning a new forward model that predicts the manipulated sensory feedback. Subsequent removal of the manipulation should then lead to mismatch in the presence of unmanipulated optic flow. In the present study, we varied the stimuli every approximately 20–30 min, which would have prevented sustained motor learning during a period that contained only a handful of majority-span trajectories.

The ability to make fine adjustments to forward flight speed may be a common feature of flying insects and hummingbirds, but reduced in other birds. Although birds can vary flight speed during different behaviours, such as migration and foraging [45,46], most birds prefer to fly using a narrow range of forward velocities near the minima of their power curves [47]. By contrast, hummingbirds have finer control of their forward flight velocity. For each of the 11 individuals in the current study, forward flight velocity varied from around 0.5 to 4.5 m s^–1^. Hummingbirds are capable of flying much faster, well over 10 m s^–1^ in wind tunnels [48] or in the field [49], but do generally fly slower through narrow passages [50]. Moreover, hummingbirds can easily transition from forward flight to hovering, which we observed an average of 2 to 8 times per tunnel transit, depending on treatment (electronic supplementary material, figure S2). Evidence for fine control of forward flight speed has also been observed in flies [8,9] and in honeybees [7]. By contrast, visual guidance experiments with budgerigars indicate that they have less fine control of their forward flight velocity [20]. This can be seen through two types of optic flow manipulations. First, a tunnel that has constant bar size, but narrows, will cause an increase in optic flow velocity as the animal gets physically closer to the walls. This manipulation causes a matched change in honeybee velocity [51], but, in budgerigars, causes a shift from a faster velocity (10 m s^–1^) to a slow speed (5 m s^–1^). Second, when vertical gratings are moving forward, budgerigars increase their speed, but only by approximately 7%. When the gratings are moving backwards, the budgerigars decrease the speed on average by only approximately 2%. Thus, results so far with budgerigars indicate that they may have one mode of faster forward flight and another of slow forward flight [20]. Hummingbirds, in contrast, have the capacity to adjust forward flight speed to a much greater extent. It is possible, however, that hummingbirds flying at faster speeds than were observed in our flight tunnel may employ additional control strategies.

Fine control of velocity by hummingbirds underlies a variety of behaviours. A single individual will use hovering flight to forage for nectar and for arthropods [52], and forward flight for movements between patch or migration sites [53]. Interspecific comparisons also reveal contexts in which different flight speeds are employed in different foraging strategies, such as during territorial encroachment (sneaking) or defence [54,55], and in different sexual displays [56]. All these behaviours require precise visuomotor control. Motor output for flight in hummingbirds is largely dictated by activation of the pectoralis major and supracoracoideus, with activation frequency controlling wingbeat frequency and activation (electromyogram) amplitude determining wing stroke amplitude [57,58]. Finer wing shape control is also evident from manoeuvres and other challenges [59,60], but how this variation is controlled by the intrinsic wing musculature is currently unresolved [61]. The results of the present study indicate two mechanisms of visual control: (1) optomotor responses driven by direct sensory feedback to control altitude during forward flight and position during hovering, and (2) use of an internal forward model to predict forward flight velocity and evaluate its consistency with actual sensory feedback.

## Data Availability

The trajectory data and all code used for analysis are available via Figshare (https://doi.org/10.6084/m9.figshare.23535717.v1) [62]. Supplementary material is available online [63].
